# Refined Requirements for Protein Regions Important for Activity of the TALE AvrBs3

**DOI:** 10.1371/journal.pone.0120214

**Published:** 2015-03-17

**Authors:** Tom Schreiber, Anika Sorgatz, Felix List, Doreen Blüher, Sabine Thieme, Matthias Wilmanns, Ulla Bonas

**Affiliations:** 1 Institute for Biology, Department of Genetics, Martin Luther University Halle-Wittenberg, Halle (Saale), Germany; 2 European Molecular Biology Laboratory, Hamburg Unit, Notkestraße 85, Hamburg, Germany; Saint Louis University, UNITED STATES

## Abstract

AvrBs3, the archetype of the family of transcription activator-like (TAL) effectors from phytopathogenic *Xanthomonas* bacteria, is translocated by the type III secretion system into the plant cell. AvrBs3 localizes to the plant cell nucleus and activates the transcription of target genes. Crucial for this is the central AvrBs3 region of 17.5 34-amino acid repeats that functions as a DNA-binding domain mediating recognition in a “one-repeat-to-one base pair” manner. Although AvrBs3 forms homodimers in the plant cell cytosol prior to nuclear import, it binds DNA as a monomer. Here, we show that complex formation of AvrBs3 proteins negatively affects their DNA-binding affinity *in vitro*. The conserved cysteine residues at position 30 of each repeat facilitate AvrBs3 complexes via disulfide bonds *in vitro* but are also required for the gene-inducing activity of the AvrBs3 monomer, i.e., activation of plant gene promoters. Our data suggest that the latter is due to a contribution to protein plasticity and that cysteine substitutions to alanine or serine result in a different DNA-binding mode. In addition, our studies revealed that extended parts of both the N-terminal and C-terminal regions of AvrBs3 contribute to DNA binding and, hence, gene-inducing activity *in planta*.

## Introduction

Transcription activator-like effectors (TALEs) comprise a large family of bacterial type III effectors, which contains important virulence factors and is restricted to phytopathogens of the genus *Xanthomonas*, with more distant orthologs in *Ralstonia solanacearum* [1,2]. Recently, three *Burkholderia rhizoxinica* genes were identified that encode proteins with weak similarity to the TALE DNA-binding domain [3,4]. TALEs are translocated into the plant cell cytosol via the *Xanthomonas* type III secretion (T3S) system and enter the nucleus, where they specifically bind to DNA and induce plant gene transcription [1]. The type member of the TALE family, AvrBs3, was isolated in 1989 from certain *X*. *campestris* pv. *vesicatoria* (*Xcv*) strains based on its ability to induce the hypersensitive response (HR), a rapid, localized cell death, in *Bs3* resistant pepper plants [5]. More recent studies showed that the HR induction is due to the AvrBs3-mediated activation of the *Bs3* resistance gene, which encodes an executor of cell death and is, in resistant pepper plants, among the *UPA* (up-regulated by AvrBs3) genes that are specifically induced by AvrBs3 [6–8]. In susceptible pepper and tomato plants, AvrBs3 causes hypertrophy, i.e., an enlargement of mesophyll cells, which is due to the induction of the cell size regulator *UPA20*, a bHLH transcription factor [6,9].

TALEs from *Xanthomonas* spp. share a highly conserved tripartite protein structure. The N-terminal region (NTR) of TALEs harbors the T3S and translocation signals required for transport into the plant cell. The C-terminal region (CTR) contains nuclear localization signals (NLSs) and an acidic activation domain (AD), both required for protein activity [1]. The most remarkable protein part, however, is the central region which is composed of nearly identical tandem repeats of typically 34 amino acids (aa) which mediate specific DNA binding. The repeat number varies among TALE proteins with most TALEs containing 15.5 to 19.5 repeats [1]. DNA binding specificity is conferred by two polymorphic amino acids at positions 12 and 13 of each repeat, termed repeat variable diresidue (RVD), which mediates binding to DNA in a "one-repeat-to-one base pair" recognition mode [10,11]. X-ray studies of *Xanthomonas* TALEs revealed that each repeat is composed of two α-helices comprising aa residues 3 to 11 and 14/15 to 33, respectively, which are connected by a short RVD-containing loop that faces the DNA [12,13]. The second residue of the RVDs (position 13) mediates direct contact to the major-groove nucleotide of the sense DNA strand, whereas the first RVD residue (position 12) stabilizes the conformation of the RVD loop [12,13]. Adjacent repeats are linked by an “outer” loop that is oriented away from the DNA. The whole repeat region forms a right-handed, superhelical structure that is wrapped around the DNA duplex tracking along the sense strand. Interestingly, the canonical TALE repeats are preceded by four non-canonical repeats (termed -3 to 0) that contribute to DNA binding [14].

Recently, TALEs gained increasing importance in biotechnological applications. The modular TALE structure and the simple DNA recognition mode of the repeats, together with sophisticated Golden Gate cloning strategies [15], e.g., the Golden TAL technology [16], allow the construction of custom-made DNA binding domains that can be combined with a variety of protein functions. TALE repeat scaffold fusions to transcription activation or repression domains enable their utilization as transcriptional modifiers in different eukaryotes. Furthermore, the repeats can be fused to enzymatic domains, as in TALE nucleases (TALENs) and recombinases (TALERs), thus creating powerful tools for genome editing [17,18]. To keep protein sizes manageable, considerable efforts have been made to minimize the TALE scaffold without suffering from substantial activity losses [17]. Therefore, it is of particular interest to determine the minimal regions of NTR and CTR required for efficient DNA binding.

In addition to specific DNA targeting, the repeats are involved in intermolecular interactions between TALE proteins. We showed previously that AvrBs3 dimerizes in the plant cell cytoplasm prior to nuclear import and that the dimerization depends on the repeat region [19]. Similarly, TALEs from *X*. *citri* formed homo- and heterodimers in yeast [20]. Here, we analyzed the mode of AvrBs3 dimerization in more detail. We show that the conserved cysteine residues at position 30 of each repeat, i.e., near the solvent-exposed outer loop, form disulfide bridges thus mediating AvrBs3 complexes *in vitro*. Interestingly, the cysteines are also required for the gene-inducing activity of the AvrBs3 monomer *in planta*. In addition, we demonstrate that the AvrBs3 NTR and CTR regions involved in DNA binding and required for full gene-inducing activity are larger than reported previously.

## Results

### AvrBs3 interacts with itself via disulfide bonds but binds DNA as a monomer

**Fig 1:**
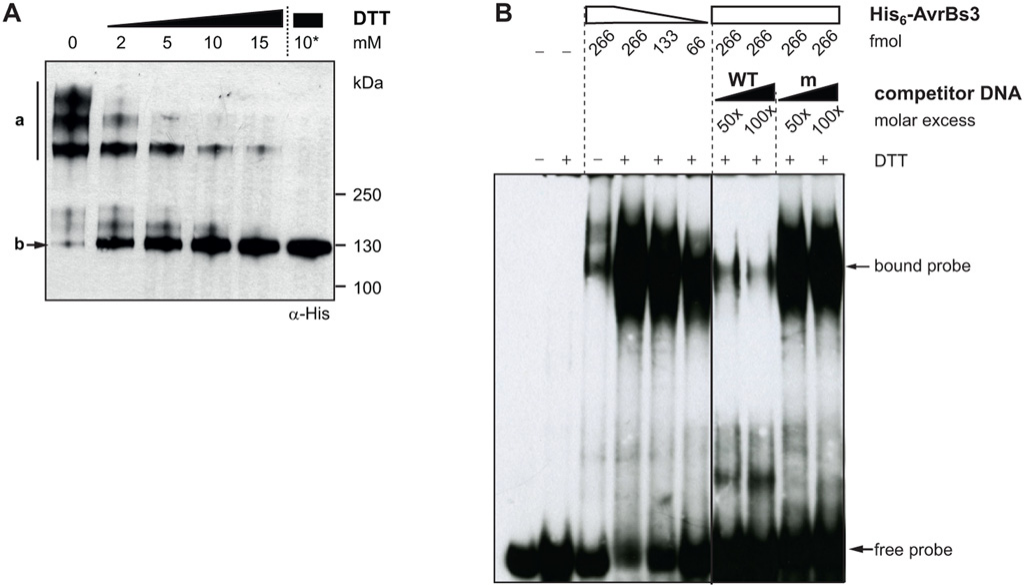
AvrBs3 complex formation interferes with DNA binding. (**A**) AvrBs3 dimerizes via disulfide bonds. 0.5 μg purified His_6_::AvrBs3, treated with different DTT concentrations for 1 h at room temperature (RT) or with 10 mM DTT overnight at 8°C (10*), was separated by a non-reducing SDS-polyacrylamide gel and analyzed by immunoblot with an α-His antibody. a, multimeric His_6_::AvrBs3; b, monomeric His_6_::AvrBs3. (**B**) AvrBs3 binds to DNA as a monomer. Electromobility shift assay (EMSA) using 333 fmol biotin-labeled 36-bp DNA-fragments derived from the *UPA20* promoter (WT) as a probe. The DNA was incubated with untreated or DTT-treated His_6_::AvrBs3 (+, 10 mM DTT overnight). Unlabeled WT and mutant ubm2 fragments (m, [8]) were used as competitor DNA. The experiments were repeated at least once with similar results.

We reported previously that AvrBs3 dimerizes dependent on the repeat region [19]. To characterize this interaction in more detail we purified recombinant His_6_-tagged AvrBs3 protein from *E*. *coli* and performed non-reducing SDS-PAGE analysis. Under non-reducing conditions most AvrBs3 molecules were present in high-molecular mass complexes (Fig. 1A). To determine the mode of AvrBs3 complex formation we incubated the purified protein with different reagents. Preliminary tests showed that addition of detergents (CHAPS, NP-40), chelating (EDTA), chaotropic (MgCl_2_, urea) and kosmotropic agents (KCl) had no effect (data not shown), whereas the reducing agent DTT dissociated the AvrBs3 protein complexes (Fig. 1A). Overnight treatment of purified AvrBs3 protein with 10 mM DTT at 8°C led to a complete dissociation of the complexes to AvrBs3 monomers (Fig. 1A) suggesting that AvrBs3 dimerization is mediated via disulfide bonds. Each repeat contains a single cysteine residue at position 30 which fits well to the observation that the repeat region is required for complex formation [19]. In addition, there are two cysteine residues in the C-terminal region of AvrBs3, which could contribute to intermolecular interactions.

To compare the DNA-binding ability of the AvrBs3 complex and its monomeric form, we performed electrophoretic mobility shift assays (EMSA). For this, we used a double-stranded biotin-labeled 36-bp DNA fragment derived from the *UPA20* promoter carrying in its center the 19-bp *UPA* box that is specifically bound by AvrBs3 [6]. We tested purified untreated (mostly multimeric) and DTT-treated (monomeric) AvrBs3 protein for protein-DNA-complex formation. While only small amounts of DNA were shifted in presence of untreated AvrBs3 protein, DTT-treatment of AvrBs3 resulted in a strongly increased amount of protein-DNA-complexes (Fig. 1B). Competition with unlabeled wild-type (WT) and mutated DNA fragments (ubm2 [8]) confirmed the specificity of the AvrBs3-DNA interaction. Our results show that AvrBs3 binds the DNA as a monomer and suggest that AvrBs3 complex formation inhibits DNA binding. This finding is in agreement with 3D data in which one TALE protein molecule binds to one DNA molecule [12,13,21].

### The cysteines in the repeat region are essential for the *in planta* activity of AvrBs3

**Fig 2:**
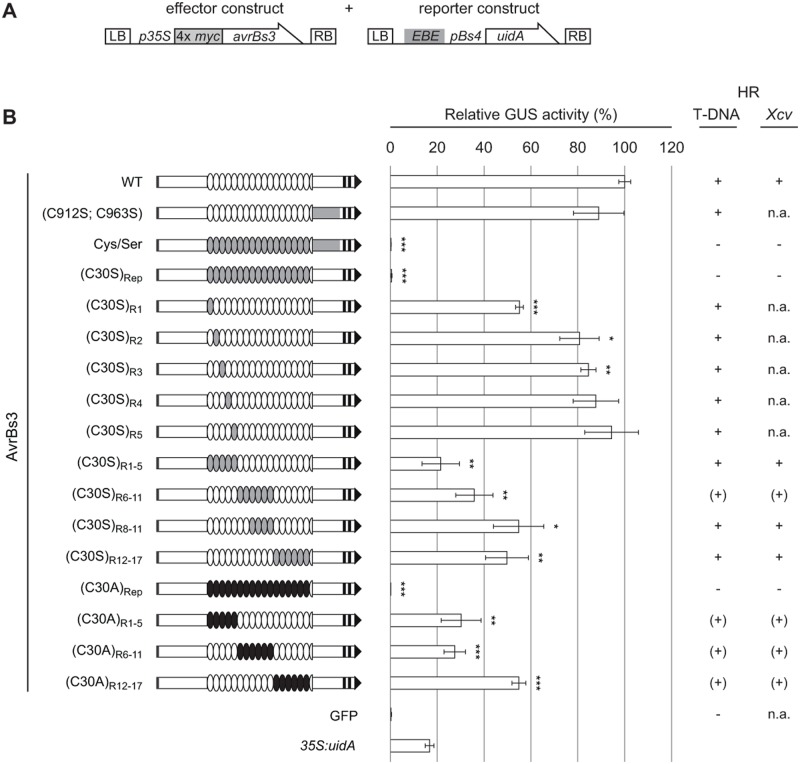
Cysteines in AvrBs3 repeats are required for target gene induction. (**A**) Constructs used for *Agrobacterium*-mediated T-DNA delivery into leaves of *N*. *benthamiana*. The effector construct allows expression of 4× c-Myc-tagged AvrBs3 derivatives under control of the *35S* promoter. The reporter construct contains the 19-bp effector binding element (EBE) of AvrBs3 in front of the tomato *Bs4*-minimal promoter driving the *uidA* (GUS) reporter gene [47]. LB, left border; RB, right border. (**B**) GUS activity was determined in *N*. *benthamiana* leaves three days after *Agrobacterium*-mediated co-delivery of the reporter construct with silencing inhibitor *p19* and effector constructs encoding AvrBs3 or derivatives. GFP served as negative, *35S*:*uidA* as positive control. Gene-inducing activities of the AvrBs3 derivatives were determined relative to the GUS activity induced by the WT AvrBs3 protein (set to 100%). Grey box, T3S signal; black boxes, NLSs; black arrow, AD. White ovals represent WT repeats, grey and black ovals denote repeats with C30S and C30A substitutions, respectively. The C-terminal region of AvrBs3 containing C912S and C963S substitutions is indicated in grey. Errors bars indicate standard deviations (SD). Asterisks indicate statistically significant differences to GUS activity induced by WT AvrBs3 (*t*-test; *, *P* < 0.05; **, *P* < 0.01; ***, *P* < 0.001). The experiment was repeated once, and four times using the *UPA20* box instead of *EBE*_AvrBs3_, with similar results. Columns on the right-hand side summarize the results of the HR induction assays in leaves of resistant pepper (ECW-30R) and *Bs3*-transgenic *N*. *benthamiana* plants after *Agrobacterium*-mediated delivery (“T-DNA”), and in pepper ECW-30R plants after inoculation of *Xcv* expressing AvrBs3 and a subset of cysteine mutants, respectively. HR development was monitored three to five dpi. +, HR three dpi; (+), delayed/partial HR five dpi;-, no HR five dpi; n.a., not analyzed. The experiments were repeated three times.

Because the AvrBs3 protein complex lacks efficient DNA-binding activity (Fig. 1B), dimerization might reduce AvrBs3 activity as transcriptional activator in the plant cell. We wondered whether AvrBs3 proteins which are unable to interact with each other and, thus, are only present in the monomeric form, display a higher activity, e.g., in reporter gene induction *in planta*. Therefore, we generated an AvrBs3 mutant derivative, AvrBs3(Cys/Ser), in which all 19 cysteine residues were substituted by the structurally related serine to minimize effects on the overall protein structure. The *in planta* activity of AvrBs3(Cys/Ser) was analyzed in quantitative β-glucuronidase (GUS) reporter assays. Two constructs were introduced into *Nicotiana benthamiana* leaves by *Agrobacterium*-mediated T-DNA delivery, one carrying the *uidA* reporter gene under control of an AvrBs3-responsive promoter and the second harboring different *avrBs3* derivatives under control of the constitutive *35S* promoter. AvrBs3(Cys/Ser) and AvrBs3(C30S)_Rep_, in which only the 17 cysteines in the repeat region were substituted to serine, failed to induce the reporter gene (Fig. 2). While the expression of both c-Myc-tagged AvrBs3 mutant variants was barely detectable in immunoblot analyses (S1A Fig.), we obtained similar reporter gene data with corresponding GFP-AvrBs3 fusion proteins (S1B Fig.) which were more stably expressed (S1C Fig.). In addition, the same result was obtained with an AvrBs3 variant in which the cysteines in the repeats were substituted by alanine [AvrBs3(C30A)_Rep_] corroborating the importance of the cysteines for AvrBs3 *in planta* activity (Fig. 2; S1B Fig.). By contrast, substitution of the two cysteines in the C-terminal region of AvrBs3 (C912S and C963S) had almost no effect on gene-inducing activity (Fig. 2). Furthermore, single cysteine substitutions in AvrBs3 repeats hardly affected reporter gene activation. However, if blocks of four to six repeats contained cysteine-to-serine or-alanine exchanges, reporter gene activation strongly decreased(Fig. 2; S1A Fig.). Our GUS reporter data correspond well to the biological activity of AvrBs3 and the cysteine mutants, i.e., the HR induction in resistant pepper (*Bs3*) and *Bs3*-transgenic *N*. *benthamiana* plants (Fig. 2). This was also observed when the proteins were delivered via the *Xcv* T3S system, i.e., under natural conditions (Fig. 2). Immunoblot analyses showed comparable expression levels of AvrBs3 cysteine mutants in *Xcv* (S1D Fig.) suggesting that the functional loss of AvrBs3(Cys/Ser) and AvrBs3(C30S)_Rep_ is not due to lower protein levels. Notably, all mutant derivatives still localized to the nucleus (S2 Fig.). Taken together, substitution of all cysteines in the TALE repeats led to a complete loss of protein function.

### Cysteine-to-serine substitutions in the AvrBs3 repeats do not significantly alter the secondary protein structure

To exclude that the loss of function of AvrBs3(C30S)_Rep_ is due to an altered secondary structure, we analyzed derivatives of AvrBs3 and AvrBs3(C30S)_Rep_, both lacking the N-terminal 152 aa, by circular dichroism (CD) spectroscopy. We reported previously that ΔN152 contains the effector domain triggering the HR in resistant pepper plants [22]. Furthermore, in our hands, ΔN152, commonly used as a scaffold for TALENs [17], is more stable *in planta* and *in vitro* than full-length AvrBs3 and can be purified to high yields. As shown in S3A Fig., ΔN152 and the cysteine mutant derivative ΔN152(C30S)_Rep_ display the same overall structure. Both proteins show α-helically dominated CD spectra with characteristic minima at 208 nm and 222 nm. However, the molar ellipticity of both spectra slightly differs in intensity, which could indicate a minimally different α-helical content of ΔN152(C30S)_Rep_ as compared to ΔN152.

### AvrBs3(C30S)_Rep_ shows reduced protein-protein interaction but retains specific DNA binding activity *in vitro*

**Fig 3:**
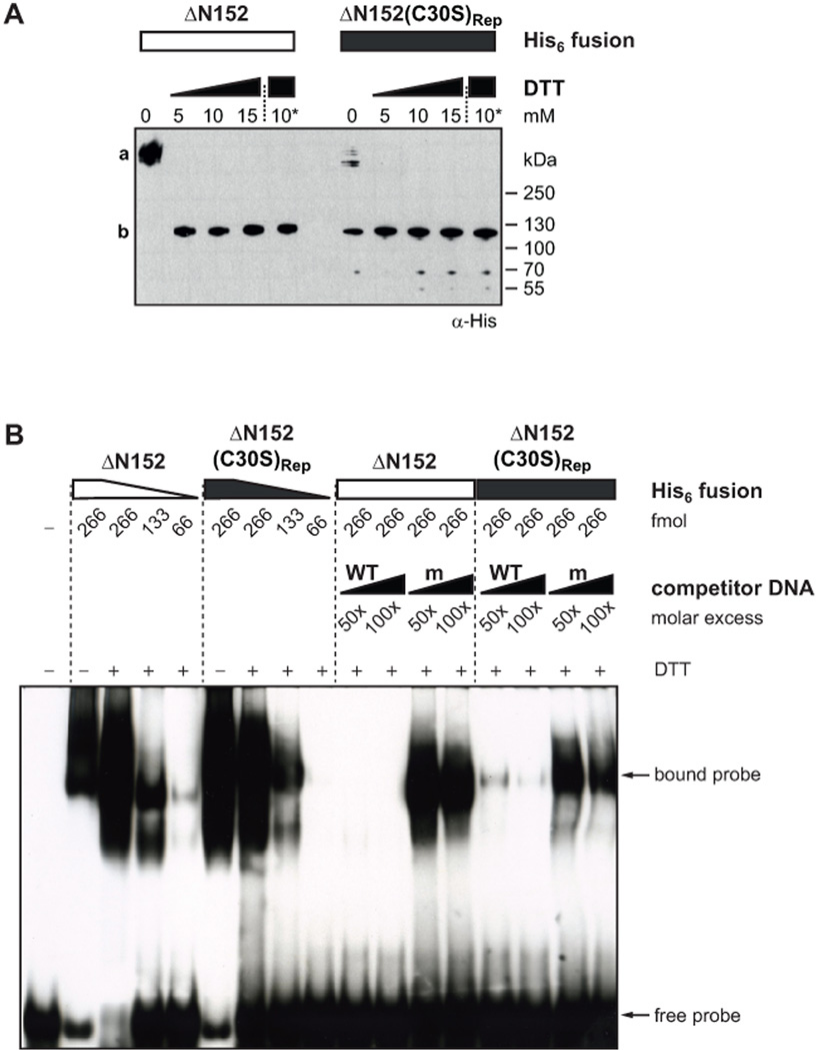
The AvrBs3 cysteine mutant is monomeric and binds specifically to DNA *in vitro*. (**A**) 0.5 μg purified His_6_::AvrBs3ΔN152 and His_6_::AvrBs3ΔN152(C30S)_Rep_, respectively, were treated with different concentrations of DTT for 1 h at RT or with 10 mM DTT overnight at 8°C (10*). Samples were separated by a non-reducing SDS-polyacrylamide gel and analyzed by immunoblot with an α-His antibody. a, multimeric His_6_::AvrBs3ΔN152; b, monomeric His_6_::AvrBs3ΔN152. (**B**) EMSA. 333 fmol biotin-labeled 36-bp DNA-fragments derived from the *UPA20* promoter were incubated with reduced His_6_::AvrBs3ΔN152 and His_6_::AvrBs3ΔN152(C30S)_Rep_ (+, incubation with 10 mM DTT overnight). Unlabeled WT and mutant ubm2 DNA fragments (m, [8]) were used as competitor DNA. The experiments were repeated at least twice with similar results.

Next, we investigated whether the complete loss of AvrBs3(C30S)_Rep_ activity *in planta* is accompanied by a lack of AvrBs3 dimerization and/or DNA binding *in vitro*. To this end, we purified ΔN152 and ΔN152(C30S)_Rep_ His_6_ fusion proteins from *E*. *coli* and analyzed protein complex formation and specific DNA binding by non-reducing SDS-PAGE and EMSA. Compared to ΔN152, ΔN152(C30S)_Rep_ protein solutions contained high amounts of monomers even without DTT (Fig. 3A). The small amounts of ΔN152(C30S)_Rep_ complexes are probably due to the two cysteines in the C-terminal region of AvrBs3. Multimeric AvrBs3ΔN152(C30S)_Rep_ could be entirely reduced to monomers by small amounts of DTT (Fig. 3A). Thus, substitution of the cysteine residues in all repeats led to a significantly reduced complex formation of AvrBs3 *in vitro*. Interestingly, ΔN152(C30S)_Rep_, bound DNA in EMSA with only slightly decreased affinity compared to ΔN152 (Fig. 3B). DNA binding was specific as determined by competition with an excess of unlabeled WT and mutant DNA, although the affinity of ΔN152(C30S)_Rep_ to unspecific DNA (ubm2 [8]) appeared to be higher compared to ΔN152 (Fig. 3B). We performed the same experiments with the ΔN152(C30A)_Rep_ derivative which behaved identically to ΔN152(C30S)_Rep_ (S4 Fig.). Taken together, substitution of all cysteine residues in the repeats to serine or alanine led to a strongly reduced AvrBs3 complex formation, but not to a loss of specific DNA binding *in vitro*.

### The AvrBs3 cysteine mutant lacks specific DNA-binding *in planta*

**Fig 4:**
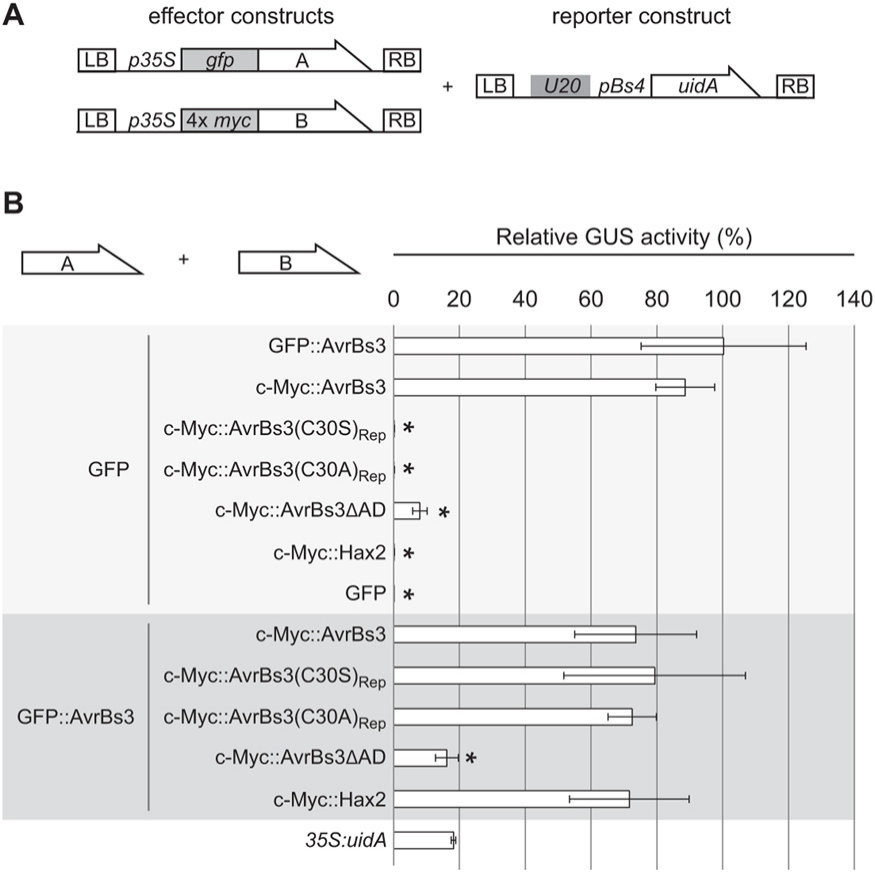
AvrBs3 cysteine mutants do not compete with AvrBs3 for DNA binding *in planta*. (**A**) T-DNA constructs used. Two different effector constructs, A and B, allow expression of (A) GFP- or (B) 4× c-Myc tagged AvrBs3 and derivatives under control of the *35S* promoter. The reporter construct contains the 19-bp *UPA20 UPA* box in front of the tomato *Bs4* minimal promoter driving a promoterless *uidA* reporter gene [48]. LB, left border; RB, right border. (**B**) GUS activity was determined in *N*. *benthamiana* leaves three days after *Agrobacterium*-mediated co-delivery of the reporter construct with the *avrBs3*- or *gfp* construct (effector construct A) and effector construct B encoding one of the indicated proteins. Values are displayed relative to the GUS activity induced by GFP and WT AvrBs3. Error bars indicate SD. *35S*:*uidA* served as control. Asterisks indicate statistically significant differences to the GUS activity induced by GFP and WT AvrBs3 (*t*-test, *P* < 0.05). The experiment was repeated twice with similar results.

Since AvrBs3(C30S)_Rep_ failed to activate the reporter gene, but retained specific DNA-binding activity *in vitro*, we wondered whether AvrBs3(C30S)_Rep_ specifically binds to DNA *in planta*. To address this question, we performed competition assays. For this, three T-DNAs carrying the *uidA* reporter driven by an AvrBs3-responsive promoter and two effector constructs were co-delivered into leaves of *N*. *benthamiana* by agroinfection (Fig. 4). GFP expression served as a control to determine the gene-inducing activity of each effector construct “alone”. As expected, co-expression of GFP and AvrBs3 resulted in strong reporter gene activation, whereas AvrBs3ΔAD lacking the C-terminal AD [22] led to little GUS activity. Expression of the natural AvrBs3 homolog Hax2 [23] and the AvrBs3 cysteine mutants AvrBs3(C30S)_Rep_ and AvrBs3(C30A)_Rep_, together with GFP, failed to activate the reporter gene (Fig. 4).

To analyze possible dominant negative effects on AvrBs3 activity *in planta*, all proteins were co-expressed with WT AvrBs3. As expected, expression of Hax2, which recognizes a different target sequence [10], did not reduce the reporter gene induction by AvrBs3. By contrast, co-expression of AvrBs3ΔAD with WT AvrBs3 significantly lowered reporter gene expression (Fig. 4). Presumably, AvrBs3ΔAD binds to the *UPA* box, but the lack of an AD hampers transcriptional gene activation. Interestingly, co-expression of AvrBs3 with the mutant derivatives AvrBs3(C30S)_Rep_ and AvrBs3(C30A)_Rep_, respectively, did not at all affect *uidA* induction by AvrBs3 (Fig. 4). While AvrBs3(C30A)_Rep_ was well expressed (S5 Fig.), AvrBs3(C30S)_Rep_ was detectable in lower amounts. This is consistent with temperature-dependent CD data revealing that *in vitro* a truncated derivative of AvrBs3(C30S)_Rep_ is considerably less stable than its WT counterpart (S3B Fig.).

### NTR- and CTR-parts of AvrBs3 are required for gene induction

**Fig 5:**
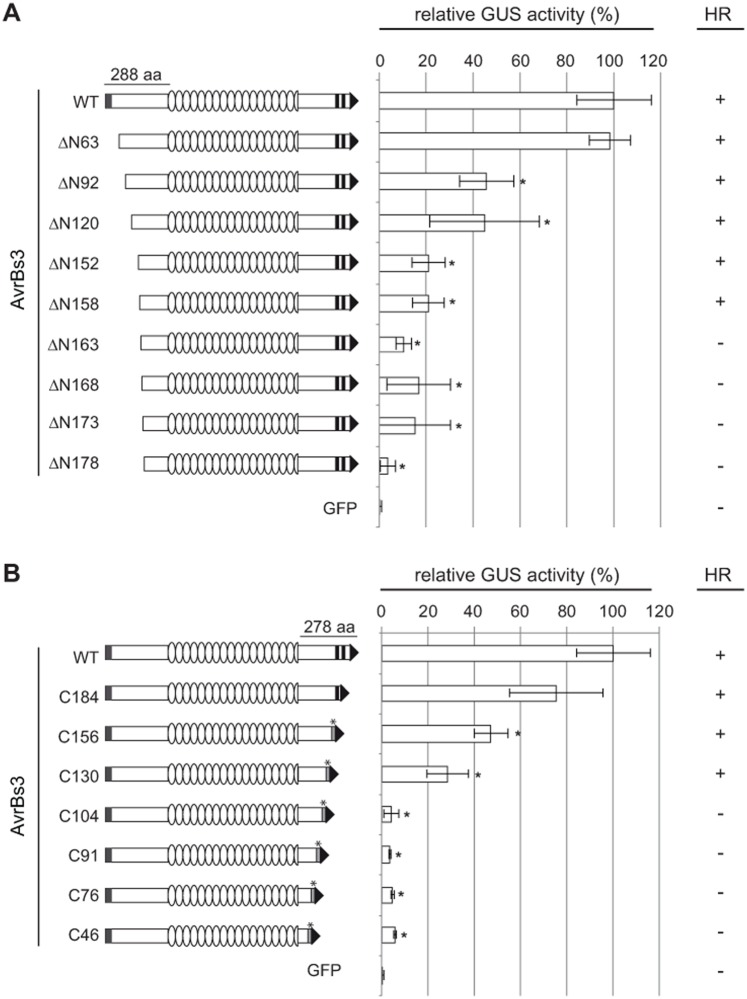
*In planta* activity of AvrBs3 N- and C-terminal deletion mutants. AvrBs3 and deletion derivatives were expressed in *N*. *benthamiana* leaves as 4× c-Myc fusions under control of the *35S* promoter by *Agrobacterium*-mediated transformation. (**A**) N-terminal deletion constructs are schematically shown on the left and are named by the number of deleted amino acids. (**B**) Names of C-terminal deletion constructs indicate the number of amino acids remaining in the C-terminal part of AvrBs3. To compensate for the loss of NLSs and AD in CTR mutants the SV40 NLS and the AvrBs3 AD were added to the new C-termini. GUS activities were determined in *N*. *benthamiana* leaves three days after *Agrobacterium*-mediated co-delivery of the reporter construct (*UPA20* box-minimal p*Bs4* driving a promoterless *uidA* [48]), the silencing inhibitor *p19*, and *avrBs3*, *avrBs3*-derivatives or *gfp* (negative control). GUS activity induced by WT AvrBs3 was set to 100%. NTR box, T3S signal; CTR boxes, AvrBs3 NLSs; box with asterisk, SV40 NLS; arrow, AD. Error bars indicate SD. Asterisks indicate statistically significant differences to the GUS activity induced by WT AvrBs3 (*t*-test, *P* < 0.05). The right column shows the HR induction by AvrBs3 and deletion derivatives in leaves of resistant pepper (ECW-30R) and *Bs3*-transgenic *N*. *benthamiana* plants three days after *Agrobacterium*-mediated delivery of the *avrBs3* or derivative constructs. The experiments were repeated three times with similar results.

Our results revealed a discrepancy between the *in vitro* and *in planta* DNA-binding ability of the AvrBs3 repeat cysteine mutants. Although the repeat region confers DNA-binding specificity, other parts of the protein might also contribute. This is consistent with recent results [6,14,24], and we wondered which parts of AvrBs3 outside of the repeat region are required for DNA binding and gene activation. The NTRs and CTRs of most natural TALEs consist of 288 aa and 278–286 aa, respectively, and are highly conserved. First, we determined the minimal length of AvrBs3 for maximal gene-inducing activity. For this, we analyzed AvrBs3 derivatives with serial N- and C-terminal deletions (Fig. 5). Due to the fact that the CTR contains the NLSs and AD, both essential for AvrBs3 *in planta* activity, we fused the SV40 NLS and the natural AvrBs3 AD to C-terminal deletion derivatives. The *in planta* activity of the resulting AvrBs3 derivatives was analyzed by GUS reporter assays in *N*. *benthamiana* leaves and the HR induction in *Bs3*-resistant pepper and *Bs3-*transgenic *N*. *benthamiana* plants. As shown in Fig. 5A, serial deletions of the AvrBs3 NTR led to a steady decrease of activity. Interestingly, the only deletion without any visible effect on AvrBs3 gene-inducing activity comprises the first 63 aa which contain the T3S and translocation signal [25]. The N-terminal deletion derivative ΔN152, which we described previously to harbor the effector domain [22] and which is commonly used as a scaffold for TALENs [17], only displayed 20% of WT activity. Deletion of the N-terminal 173 aa led to an almost complete loss of reporter gene induction and no longer reproducibly induced the HR (Fig. 5A), although the protein was well expressed (S6 Fig.).

Interestingly, all CTR-deletions tested affected the gene-inducing activity of AvrBs3. While expression of AvrBs3-C184, C156 and C130 led to a moderate loss of activity in our GUS reporter assays and induced the HR in *Bs3*-resistant pepper and *N*. *benthamiana* plants, AvrBs3-C91 and shorter versions almost completely lost the ability to induce the reporter gene and no longer reproducibly induced the HR (Fig. 5B; for protein expression see S6 Fig.). This indicates that at least 130 aa of the CTR immediately downstream of the repeats are essential for AvrBs3 *in planta* activity.

### The imperfect leucine zipper motif in the CTR has no relevance for the gene-inducing activity of AvrBs3

**Fig 6:**
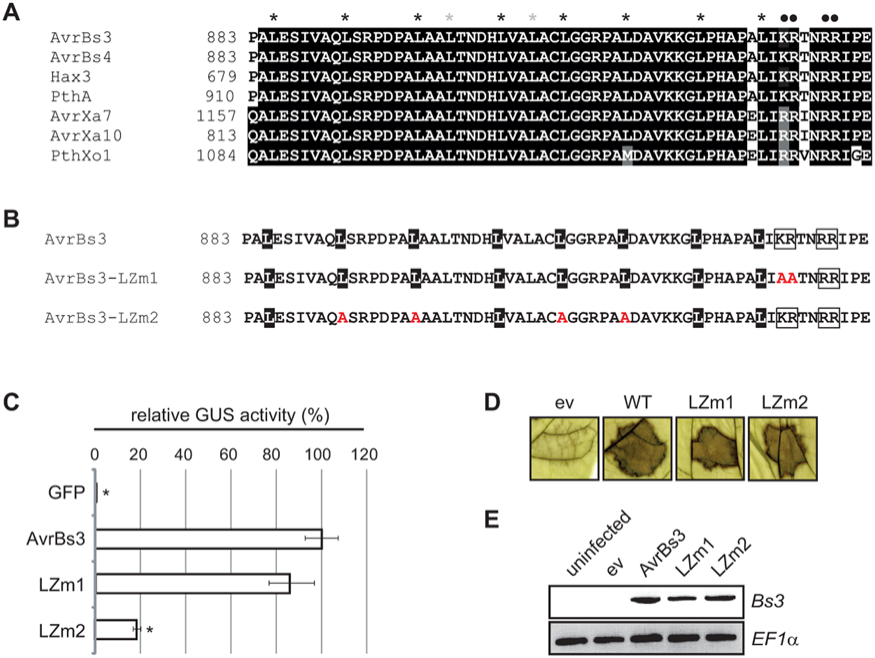
Analysis of the imperfect leucine zipper motif in AvrBs3. (**A**) Sequence comparison between the leucine zipper-like motif in the CTR of AvrBs3 and a subset of homologs using ClustalW. Identical amino acids (white letters on black background) and similar amino acids (white letters on grey background) were shaded using Boxshade. Leucines corresponding to the proposed leucine repeats were labelled with a black asterisk, leucines that do not correspond to leucine repeats with a grey asterisk and aa of the basic region with a black dot. **(B)** AvrBs3 was mutated in the basic region (AvrBs3-LZm1) and in leucines of the leucine-rich region (AvrBs3-LZm2). Leucines are given in black, the basic regions are boxed. **(C)** *UPA20* box activation by AvrBs3 and AvrBs3 mutant derivatives shown in B. GUS activities were determined in leaves of *N*. *benthamiana* three days after *Agrobacterium*-mediated co-delivery of the reporter construct (*UPA20* box-minimal p*Bs4* driving a promoterless *uidA* [48]), together with silencing inhibitor *p19* and *gfp*, *avrBs3* or *avrBs3* derivatives. GUS activities are given relative to the GUS activity induced by WT AvrBs3. Error bars indicate SD. Asterisks indicate statistically significant differences as compared to WT AvrBs3 (*t*-test, *P* < 0.05). **(D)** HR induction by AvrBs3 and mutant derivatives in *Bs3* ECW-30R plants. *Xcv* 85–10 containing pGGX1 (empty vector, ev) or pGGX1 driving expression of *avrBs3*, *avrBs3-*LZm1 and *avrBs3-*LZm2, respectively, were inoculated into leaves of *Bs3* pepper plants (ECW-30R). Leaves were harvested three dpi and bleached with ethanol to better visualize the HR. **(E)** *Bs3* gene induction by AvrBs3 and mutant derivatives in leaves of pepper ECW-30R. RT-PCR analysis 10 h after inoculation of the *Xcv* strains described in D. *EF1α* was used as control for equal cDNA amounts. RT-PCR was repeated once, the other experiments at least twice with similar results.

Interestingly, there is an imperfect leucine zipper-like motif in the 130-aa C-terminal region revealed to be essential for AvrBs3 activity *in planta* (see above) [26]. A leucine zipper usually consists of a repetition of leucines every seventh amino acid (heptads) and a basic region, and mediates protein dimerization and DNA binding [27]. However, the sequence in AvrBs3 and its homologs deviates from the leucine periodicity (Fig. 6A). To analyze whether the leucine zipper-like motif contributes to AvrBs3 gene-inducing activity, derivatives with substitutions in the basic region (AvrBs3-LZm1) and leucine residues (AvrBs3-LZm2), respectively, were generated and analyzed by GUS reporter assays (Fig. 6B, C). While AvrBs3-LZm1 induced the reporter gene comparably to WT AvrBs3, AvrBs3-LZm2 gene-inducing activity dropped to 20% (Fig. 6C). Immunoblot analysis showed that AvrBs3 and AvrBs3-LZm1 were expressed in similar amounts, whereas the AvrBs3-LZm2 expression level was reduced (S7A Fig.). To evaluate whether the AvrBs3 derivative expression level and *uidA* reporter gene induction are correlated, we tested for the HR induction and *Bs3* gene activation in resistant pepper plants after *Xcv* infection. *Xcv* T3S system delivery of AvrBs3, AvrBs3-LZm1 and AvrBs3-LZm2 triggered the HR in resistant pepper plants and induced the *Bs3* resistance gene to similar levels (Fig. 6D, E). Immunoblot analyses showed comparable expression of all three proteins in *Xcv* (S7B Fig.). These data suggest that the differences in gene-inducing activity observed in the GUS assays (Fig. 6C) are indeed due to differences in expression level rather than functional importance of the leucine residues.

### Both the NTR and CTR of AvrBs3 contribute to DNA binding

**Fig 7:**
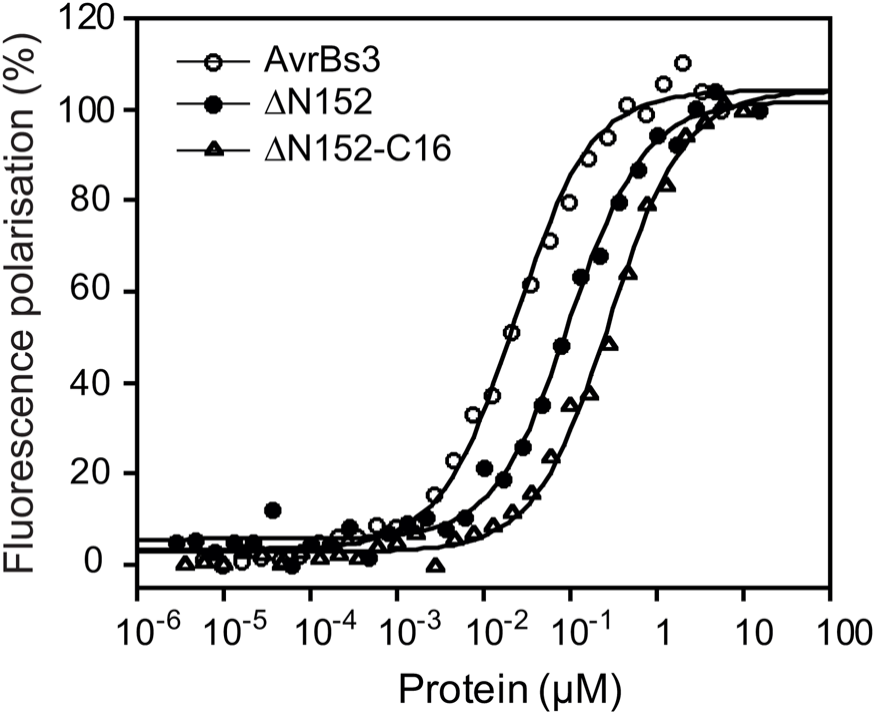
AvrBs3 NTR and CTR contribute to DNA binding to different extents. Fluorescence polarization titrations of fluorescein-labeled dsDNA by AvrBs3 and deletion mutants. Increasing concentrations of purified His_6_-tagged AvrBs3 (open circles), AvrBs3ΔN152 (closed circles) and AvrBs3ΔN152-C16 (CTR deleted, except for the first 16 aa downstream of the repeats; open squares) were incubated under reducing conditions with fluorescein-labeled 36-bp DNA fragments derived from the *UPA20* promoter. Fluorescence polarization intensities were normalized and plotted as function of protein concentration. The results are representative of three independent measurements. Dissociation constants (K_D_^app^) were determined by curve fitting with a one-site saturation and nonspecific binding model using Kaleidagraph 4.0 (Synergy Software). The K_D_^app^ for AvrBs3 is 23.2 ± 1.6 nM, and K_D_ values of 99.2 ± 7.5 nM and 282.2 ± 18.1 nM were calculated for the truncated derivatives AvrBs3ΔN152 and AvrBs3ΔN151-C16, respectively.

As shown above deletions in the NTR and CTR led to reduced AvrBs3 activity *in planta* (Fig. 5). Since binding to DNA is a prerequisite for gene induction, we quantitatively analyzed the DNA-binding affinity of selected N- and C-terminal AvrBs3 deletion derivatives by fluorescence polarization measurements. As target DNA we used the 36-bp *UPA20* promoter fragment that contained the *UPA* box [6]. Interestingly, fluorescence titrations revealed a four-fold reduction in K_D_^app^ for the N-terminally truncated construct ΔN152 (99.2 ± 7.5 nM) as compared to WT AvrBs3 (23.2 ± 1.6 nM). Further truncation of the C-terminal region as in ΔN152-C16 led to an additive decrease in affinity (282.2 ± 18.1 nM) (Fig. 7). These results suggest that both the NTR and CTR are needed for full DNA-binding affinity of AvrBs3.

## Discussion

### AvrBs3 dimerization via disulfide bridges inhibits DNA binding

Here, we demonstrated that the cysteine residues which are conserved in all canonical TALE repeats are essential for protein activity. The cysteines form disulfide bridges in the DNA-free state of AvrBs3 *in vitro*, and are required *in planta* for specific DNA-binding and gene-inducing activity. To our knowledge, this is the first study that systematically analyzed a conserved, non-RVD amino acid in TALE repeats.

Disulfide bridges between the solvent-exposed cysteine residues in the repeats play a role in intermolecular AvrBs3 interactions. Complex formation of AvrBs3 and other TALEs was shown in yeast, *in vitro* and *in planta* and depends on the repeat region [19,20]. AvrBs3 dimerizes in the plant cell cytoplasm prior to nuclear import [19]. Because DTT treatments revealed that AvrBs3 complex formation is not only irrelevant but even detrimental for target promoter binding, AvrBs3 dimers probably dissociate in the nucleus. Interestingly, AvrBs3 homologs from *X*. *axonopodis* pv. *citri* were found to interact with a thioredoxin from *Citrus sinensis* which may reduce TALE complexes to monomers *in planta* [20]. This is in agreement with the 3D structures of TALEs bound to their target DNA as well as ITC data in which one protein molecule binds one molecule of double-stranded DNA [12,13,21]. This scenario is reminiscent of the TATA-binding protein which also is imported into the nucleus as a dimer [28].

Prolonged treatment of purified AvrBs3 protein with DTT considerably improved the DNA affinity of AvrBs3 in EMSA. This represents an important technical advance for future DNA-binding studies of TALEs and respective fusion proteins and is of special importance if constructs with different repeat numbers are compared.

### Cysteines in the AvrBs3 repeats contribute to protein plasticity

Intriguingly, we found that the cysteines in the repeats are essential for AvrBs3 activity. X-ray analyses revealed conformational changes upon DNA binding of an artificial TALE, dHax3, which helps to explain our results. Strikingly, the dHax3 superhelix is compressed in its DNA-bound state compared to the free protein, i.e., the size of the repeat region (here, one superhelical turn) is reduced from 60 Å to 35 Å [13]. It was postulated that the flexibility of the repeat domain is due to van der Waals interactions between adjacent repeats, which tolerate minor distance shifts [13]. Notably, upon DNA binding of TALEs the structural alterations in each repeat are relatively small, but with increasing repeat numbers they result in pronounced conformational changes [13]. The conserved cysteine residue at position 30 in each repeat is located in the second helix near the outer loop that is positioned away from the DNA [12,13]. Since our CD spectroscopy data suggest that the secondary protein structure of AvrBs3(C30S)_Rep_ is comparable to WT AvrBs3, we believe that the cysteine substitutions only slightly affect AvrBs3 plasticity. Removing the lipophilic “cysteine hinge” might change the flexibility of the outer loop which is supported by a recent X-ray study stating that the structural plasticity of the repeats is facilitated by aa residues 26–34 [29].

Considering the model that DNA binding of TALEs starts with the N-terminal protein region which acts as a “nucleation site” [14,24], we assume that the first repeats of AvrBs3(C30S)_Rep_ specifically bind to DNA, whereas more C-terminally located repeats fail to efficiently contact the corresponding DNA bases. Consequently, this changes the position of the AvrBs3 AD relative to the DNA, thus probably preventing recruitment of plant proteins necessary for gene activation.

### Most of NTR and CTR are required for AvrBs3 activity

Here, quantitative analyses of mutant AvrBs3 derivatives revealed that most of the NTR and the entire CTR contribute to its gene-inducing activity. Deletion of the N-terminal 63 aa retained the same level of transcription activity as WT AvrBs3, whereas all other deletions resulted in a substantial loss of reporter gene induction. This is consistent with data on the activity of N-terminally truncated TAL effectors in mammalian cells [30]. Similarly, TALENs containing a full-length NTR were more active compared to TALENs with the commonly used ΔN152 scaffold, at least when low protein amounts were used [31].

The five-fold reduced gene-inducing activity of AvrBs3ΔN152 is accompanied by a strong reduction in DNA affinity as determined by our fluorescence polarization measurements. We assume that the activity loss of N-terminal AvrBs3 deletion constructs is due to impaired DNA binding. It was shown previously that an AvrBs3 derivative consisting of the repeat region alone displays considerably reduced DNA binding activity as compared to the full-length protein [6]. A recent study of an artificial TALE described four degenerated repeats (designated repeat -3 to 0; aa 162–288) which precede the region of canonical repeats and are involved in DNA binding in a sequence-independent manner [14]. Taken together, the data presented here suggest an even larger DNA-binding domain in the NTR than described by Gao et al. [14].

Fluorescence titration revealed a contribution of the AvrBs3 CTR to DNA binding. This contradicts recent results by Gao et al. (2012) who performed ITC analyses with derivatives of an artificial TALE similar to our constructs, but found no contribution of the CTR to DNA binding [14]. Recently, two degenerated repeats (termed +1 and +2) were predicted in the CTR downstream of the canonical repeat region [32], which might contribute to DNA binding. The CTR of TALEs contains the NLSs and AD which are essential for nuclear import and activation, respectively, and is generally thought to serve as interaction platform for host proteins. Here, the first 130 aa adjacent to the repeats turned out to be essential for target gene induction. Interestingly, they contain a leucine-rich region, described as “imperfect leucine zipper” [26]. Although mutations we introduced had no measurable effect on AvrBs3 gene-inducing activity, it is possible that this repeat-proximal region mediates the interaction with plant proteins. Indeed, the “leucine-rich repeat” of the TALE PthA2 was recently described as interaction surface for an HMG protein from *Citrus* [33]. However, the necessity of the leucine residues was not addressed yet using PthA2 mutant derivatives.

### Conclusions

Taken together, the conserved cysteine residues in the AvrBs3 repeats are essential for protein-DNA interactions and, hence, AvrBs3 gene-inducing activity. The contribution of the cysteines to AvrBs3 plasticity is probably required for the conformational changes during DNA binding. Furthermore, we demonstrated that large NTR- and CTR-parts of AvrBs3 contribute to DNA affinity. These findings and the optimized conditions for DNA-binding assays will help to improve the generation of TALENs, TALERs and other applications using the TALE scaffold to target specific DNA sequences.

## Materials and Methods

### Plant material and inoculations

*Nicotiana benthamiana* and pepper (*Capsicum annuum*) cultivar ECW-30R (*Bs3*) plants were grown in the greenhouse under standard conditions (day and night temperatures of 23°C and 19°C, respectively, for *N*. *benthamiana*, and 25 and 19°C for pepper, with 16 h light and 40 to 60% humidity). For *in planta* expression studies, mature leaves of five- to seven-week-old plants were inoculated with *Agrobacterium tumefaciens* adjusted in infiltration buffer to OD_600_ = 0.8 and inoculated using a needleless syringe as described [34]. *Xcv* was inoculated (6×10^7^ cfu/ml in 10 mM MgCl_2_) into leaves of six-week-old pepper plants using a needleless syringe.

### Bacterial strains and growth conditions

If not stated otherwise, *Escherichia coli* BL21(DE3) (Agilent Technologies Inc., Santa Clara, USA) and TOP10 (Life Technologies GmbH, Darmstadt, Germany) were cultivated at 37°C in LB (lysogeny broth) medium [35], *A*. *tumefaciens* GV3101 [36] and derivatives were grown at 30°C in YEB (yeast extract broth) and *Xcv* strain 85–10 [37] and derivatives in NYG (nutrient yeast glycerol) [38] supplemented with appropriate antibiotics. Plasmids were introduced into *E*. *coli* and *A*. *tumefaciens* by electroporation, and into *Xcv* by conjugation using pRK2013 as helper plasmid in triparental matings [39].

### Generation of Golden Gate expression vectors

The binary vectors pGGA2 and pGGA8 are based on the pBGWFS7 backbone [40] and allow *in planta* expression of genes 5´-translationally fused to GFP and 4× c-Myc, respectively, under the control of the cauliflower mosaic virus *35S* promoter. The broad host range vector pGGX1 contains the backbone of pBBR1MCS-5 [41] and allows the expression of genes in *Xanthomonas* 3´-translationally fused to a FLAG epitope under control of the *lac* promoter. The *E*. *coli* expression vector pGGE6 contains the backbone of pQE60 (QIAGEN, Hilden, Germany), in which the selectable marker (ampR) was exchanged by kanamycin resistance. The gene of interest is expressed 5´-translationally fused to a hexa-histidine (His_6_) tag under control of p*T7*. All vectors contain the chloramphenicol resistance-*ccdB* cassette from pGWB2 [42]. To allow cloning of DNA fragments by *Bsa*I cut-ligation [15], additional *Bsa*I restriction sites in the plasmids were removed. Cloning details are available upon request.

### Construction of AvrBs3 derivatives and controls

Generally, DNA fragments were amplified using oligonucleotides providing *Bsa*I sites and Phusion polymerase (New England Biolabs GmbH, Frankfurt/Main, Germany). All cloned fragments were sequenced. Oligonucleotides are listed in S1 Table. AvrBs3 and derivatives were cloned in a two-step cut-ligation reaction using the Golden TAL Technology [16]. DNA fragments encoding the NTR (864 bp) and CTR of AvrBs3 (837 bp, divided into 467 bp and 370 bp fragments) were amplified and cloned into pJet1.2 (Fisher Scientific GmbH, Schwerte, Germany). The repeat region of *avrBs3* (1794 bp) was divided into three modules each consisting of six single-repeat submodules. The submodules were synthesized by GeneArt (GeneArt AG, Regensburg, Germany), flanked by *Bpi*I sites, and cloned into pMK. In the first step of *avrBs3* construction, six-repeat modules were assembled by *BpiI* cut-ligation of single repeat submodules into pUC57-derived vectors (pUC57-D1–6, pUC57-D7–12 and pUC57-D13–17.5), flanked by *Bsa*I sites. Then, the three six-repeat modules and the modules encoding the NTR and CTR parts of AvrBs3 were ligated into respective expression vectors by *Bsa*I cut-ligation [15]. Mutations in the *avrBs3* coding sequence were introduced into the respective modules by site-directed mutagenesis before assembly. To create *avrBs3ΔAD* and truncated CTR modules, corresponding fragments were amplified, cloned into pJet1.2 and assembled with the NTR and repeat modules in pGGA8. To compensate for the loss of NLS and AD in C-terminally truncated AvrBs3 derivatives, fragments encoding the SV40 NLS [43] and the AvrBs3 AD were added at the 3´ end. For *hax2*, gene fragments encoding the NTR, the repeat region and the CTR were amplified separately and cloned as described above. *gfp* was amplified and cloned into pGGA8. For empty vector controls, a pair of annealed oligonucleotides (S1 Table) was cloned into pGGA2 and pGGX1.

### β-glucuronidase (GUS) assay

Three *N*. *benthamiana* plants per experiment were inoculated with *Agrobacterium* derivatives. Three *Agrobacterium* strains delivering a promoter construct, a TAL expression construct (pGGA8 derivative) and the silencing inhibitor *p19* (plasmid pBin61:p19 [44]), respectively, were adjusted to OD_600_ = 0.8 and mixed in a 1:1:1 ratio. p19 was not used in competition assays (Fig. 4). Two leaf discs (0.9 cm diameter) per plant were sampled three days post infiltration (dpi) and GUS activities determined as described [6].

### Microscopy

For subcellular localization of GFP and respective fusions (pGGA2 derivatives) lower epidermal cells of *N*. *benthamiana* were inspected with a confocal laser scanning microscope LSM 780 and the ZEN software (Carl Zeiss GmbH, Göttingen, Germany) according to the manufacturer. To visualize nuclei, leaves were infiltrated with 0.1% (w/v) diamidine-2-phenylindol (DAPI) solution 1 h before microscopy.

### Immunoblot analysis

Protein extracts from infected *N*. *benthamiana* leaves were prepared by grinding three discs (0.9 cm diameter) from different leaves in liquid nitrogen and adding 100 μl 4× Laemmli buffer. 20 μl protein samples (2 μl for GFP) were separated on 10% SDS polyacrylamide gels and subjected to immunoblot analyses with α-c-Myc (Santa Cruz Biotechnology Inc., Dallas, USA) or α-GFP (Life Technologies) antibodies. For protein expression analysis of *Xcv*, bacteria were grown two days on agar plates, then overnight in liquid culture. 500 μl culture (OD_600_ = 0.4) were harvested and the cells resuspended in 50 μl 2× Laemmli buffer. 10 μl protein samples were separated on a 10% SDS polyacrylamide gel and subjected to immunoblot analysis with α-FLAG (Sigma-Aldrich, Taufkirchen, Germany) and α-GroEL (Stressgen, Victoria, Canada) antibodies. α-mouse Ig and α-rabbit Ig were used as secondary antibodies (GE Healthcare Bio-Sciences, Pittsburgh, USA). Antibody reactions were visualized by enhanced chemiluminescence (GE Healthcare).

### RT-PCR analysis

ECW-30R pepper plants were inoculated with derivatives of *Xcv* strain 85–10 (OD_600_ of 0.05) expressing *avrBs3* and mutant derivatives. RNA was extracted from ten leaf discs (diameter 0.28 cm), harvested 10 hpi, using the RNeasy Plant Miniprep kit (Qiagen, Hilden, Germany). RNA concentrations were determined with an ND-1000 spectrophotometer (NanoDrop Technologies, Rockland, DE, USA). cDNA was synthesized by reverse transcription using an oligo dT-primer and the Revert Aid First Strand Synthesis Kit (Fermentas). For RT-PCR of *Bs3* the RT-PCR was performed as described using oligonucleotides Cand-7–01-fwd and Cand-7–01-rev for *Bs3* and RS-EFrt-F1 and RS-EFrt-R1 for *EF-1α* (used for RT-PCR normalization) [7].

### Protein expression in *E*. *coli* and purification

*E*. *coli* BL21(DE3) (Agilent Technologies) carrying pGGE6 derivatives were grown in TB (terrific broth) medium [45]. Expression was induced at an OD_600_ of 0.6 to 0.9 in presence of 0.5 μM IPTG for 18 h at 16°C. Cells were harvested by centrifugation, resuspended in ice-cold lysis buffer (50 mM Tris/HCl, 10 mM NaCl, 10 mM imidazol, 0.1% Tween20, pH 8.0), supplemented with protease inhibitors (Roche), and lysed by three freeze-thaw cycles. Protein extracts were separated by Ni-NTA sepharose column (QIAGEN), equilibrated with lysis buffer. After washing with lysis and wash buffer (50 mM Tris, 10 mM NaCl, 40 mM imidazol, 0.1% Tween20, pH 8.0), respectively, His_6_-tagged proteins were eluted with elution buffer (50 mM Tris, 10 mM NaCl, 250 mM imidazol, 0.1% Tween20, pH 8.0) at 4°C.

For protein purification under reducing conditions, buffers were supplemented with 1 mM Tris (2-carboxyethyl) phosphine (TCEP). Elution fractions containing enriched protein were pooled and dialysed 2× 5 h with 200× volumes of storage buffer (50 mM Tris, 10 mM NaCl, 10% glycerol, pH 8.0). Protein concentrations were determined at 280 nm using the molar absorption coefficient calculated according to Pace et al. [46], or by Bradford assay (BioRad, Hercules, USA). Protein aliquots were frozen in liquid nitrogen and stored at -80°C.

### Non-reducing SDS-PAGE

0.5 μg aliquots of His_6_-tagged proteins purified under non-reducing conditions were incubated with 0, 2, 5, 10 and 15 mM DTT for 1 h at room temperature (RT) or with 10 mM DTT overnight at 8°C, respectively. Proteins were incubated with non-reducing loading dye (100 mM Tris, 10% glycerol, 3% (w/v) SDS, 55% (v/v) 2-mercaptoethanol, 0.1 mg/ml bromphenol blue, pH 8.0) in a 1:1 ratio for 10 min at RT prior to loading onto a 4–20% gradient SDS gel (SERVA Electrophoresis GmbH, Heidelberg, Germany) followed by immunoblot analysis using α-penta-His (QIAGEN) and α-mouse Ig (GE Healthcare) antibodies.

### Electrophoretic mobility shift assay (EMSA)

Recombinant His_6_-tagged proteins were purified from *E*. *coli* under non-reducing conditions and treated with 10 mM DTT overnight at 8°C. To obtain dsDNA, complementary non-labeled and 5´-biotin-labeled oligonucleotides (Metabion GmbH, Martinsried, Germany) were annealed. Binding reactions of 0.033 nM labeled E1U20 dsDNA [6] with different concentrations of protein were performed in EMSA buffer (10 mM HEPES (pH 7.5), 100 mM KCl, 5 mM MgSO_4_, 5% glycerol) for 30 min at RT. For competition, an excess of non-labeled E1U20 [6] and E2U20 [8] dsDNA, respectively, was pre-incubated with 0.266 nM protein for 10 min at RT before labeled dsDNA was added. DNA fragments were separated on a 5% native polyacrylamide gel with EMSA running buffer (0.5× TBE, 1% glycerol) at 8°C, transferred to a positively charged nylon membrane by wet blot using 0.5× TBE buffer (30 min, constant 100V, 8°C), and fixed by 1 h baking at 100°C. Labeled DNA fragments were detected with the Chemiluminescent Nucleic Acid Detection Module (Pierce, Rockford, IL, USA) according to the manufacturer.

### Fluorescence anisotropy assays

Fluorescence anisotropy assays were performed in assay buffer (50 mM Tris/HCl pH 8.0, 150 mM NaCl, 1 mM TCEP, 0.1 mg/ml BSA) using a TECAN infinite M1000 plate reader (Tecan Group Ltd., Männedorf, Switzerland) and 480 nm excitation/520 nm emission wavelengths. His_6_-tagged AvrBs3 and variants were purified under reducing conditions and used at concentrations sufficient to saturate dsDNA E1U20 fragment [6] binding at the highest concentration. A protein dilution series (factor 0.6) was titrated with 0.5 nM fluoresceine-labeled DNA. DNA and protein mixtures were incubated in a 96-well plate for 10 min at RT before readout. Values are the means of three independent measurements wherein each measured point was set up in duplicate. Binding data were analyzed using Kaleidagraph 4.0 (Synergy Software, Reading, USA) and corrected with a linear equation for unspecific binding and using a simple model assuming one binding site per DNA fragment and a 1:1 stoichiometry. For comparison, fluorescence polarization units were normalized; 100% represents saturated binding.

### Circular dichroism (CD) spectroscopy

His_6_-tagged proteins were purified under reducing conditions and subjected to CD spectroscopy at a concentration of 5 μM in 15 mM Tris/HCl pH 8.0, 150 mM NaCl using a quartz cuvette of 0.1 cm path length in a Chirascan CD spectrometer (Applied Photophysics Ltd., Leatherhead, UK). CD-spectra were recorded between 190 and 280 nm with 1 nm intervals at 25°C. Three CD-spectra were averaged and the baseline corrected by subtraction of the buffer spectrum. Measured values in mdeg were converted to mean residue molar ellipticity. CD melting curves were recorded with a temperature slope of 1°C/min at 222 nm between 20 and 70°C. The signals were converted into the fraction of folded protein. Melting temperatures (T_m_) were calculated by curve-fit to the Boltzmann sigmoidal equation using Kaleidagraph 4.0 (Synergy Software).

## Supporting Information

S1 Fig Substitution of the cysteines in the repeat region to serine or alanine affects AvrBs3 gene-inducing activity. **(A)** *Agrobacterium*-mediated expression of AvrBs3 WT and cysteine mutants in leaves of *N*. *benthamiana*. Samples were harvested three dpi and analyzed by immunoblot using an α-c-Myc antibody. **(B)** GUS activity was determined in *N*. *benthamiana* leaves three dpi of *Agrobacterium* delivering the reporter construct (*EBE*_AvrBs3_-p*Bs4* minimal promoter driving a promoterless *uidA* [47]) and silencing inhibitor *p19*, and effector constructs containing *avrBs3* or *avrBs3*-derivatives fused to *gfp*. The GUS activity induced by each effector construct is shown relative to the GUS activity induced by WT AvrBs3. Error bars indicate SD. Asterisks indicate statistically significant differences to the GUS activity induced by WT AvrBs3 (*t*-test, *P* < 0.05). (**C**) *Agrobacterium*-mediated expression of WT AvrBs3 and the cysteine mutants in leaves of *N*. *benthamiana*. Samples were harvested three dpi from the same areas as investigated in (B) and analyzed by immunoblot using an α-GFP antibody. **(D)** Western blot analysis of *Xcv* 85–10 expressing *avrBs3* and the *avrBs3* cysteine mutants, respectively, or carrying pGGX1 (empty vector, ev). Total protein extracts were analyzed by immunoblot using a FLAG-specific antibody; α-GroEL served as control for equal protein amounts. The experiments were repeated at least once with similar results.
(EPS)

S2 Fig AvrBs3 cysteine exchange mutant proteins localize to the nucleus. **(A)** Confocal laser scanning microscopy of *N*. *benthamiana* leaves three days after *Agrobacterium*-mediated transfer of *gfp* or *gfp* fusions of WT *avrBs3*, the cysteine mutant AvrBs3(Cys/Ser) and the repeat cysteine mutants AvrBs3(C30S)_Rep_ and AvrBs3(C30A)_Rep_. DAPI (4´,6´-Diamidino-2-phenylindole) staining indicates nuclei. Scale bars, 20 μm. **(B)** *Agrobacterium*-mediated expression of GFP fusion proteins. Samples were harvested three dpi from the same areas as investigated in (A) and analyzed by immunoblot with an α-GFP antibody. The arrow indicates the expected size of the protein. The experiment was repeated three times with similar results.
(EPS)

S3 Fig Cysteine-to-serine substitutions in the AvrBs3 repeats do not significantly alter the α-helical content but affect protein stability. **(A)** Far-UV CD-spectra of AvrBs3ΔN152 (closed circles) and AvrBs3ΔN152(C30S)_Rep_ (open circles). His_6_-tagged proteins were purified under reducing conditions and subjected to CD-spectroscopy. The calculated molar ellipticity (Θ_*molar*_) is plotted as function of recorded wavelengths from 200 to 280 nm using Kaleidagraph 4.0 (Synergy Software). The curves represent the means of three measurements. **(B)** CD melting curves (20–70°C) of His_6_-tagged proteins, purified under reducing conditions, recorded at 222 nm. The fraction of folded protein for AvrBs3ΔN152-C16 (CTR deleted except for the first 16 aa downstream of the repeats; closed circles) and AvrBs3ΔN152-C16(C30S)_Rep_ (open circles) is plotted as a function of temperature. Melting temperatures (T_m_) were calculated by fitting the curves to the Boltzmann sigmoidal equation using Kaleidagraph 4.0 (R^2^ values >0.99). ΔN152-C16 has a T_m_ value of 46.8±0.08°C and ΔN152-C16(C30S)_Rep_ of 36.2±0.04°C. The experiment was repeated three times with similar results.
(EPS)

S4 Fig AvrBs3(C30S)_Rep_ and AvrBs3(C30A)_Rep_ are monomeric and specifically bind DNA *in vitro*. (**A**) 0.5 μg purified His_6_::AvrBs3ΔN152(C30S)_Rep_ and His_6_::AvrBs3ΔN152(C30A)_Rep_, respectively, were treated with different concentrations of DTT for 1 h at RT or with 10 mM DTT overnight at 8°C (10*). Samples were separated by a non-reducing SDS-PAGE and analyzed by immunoblot with an α-His antibody. (**B**) EMSA using 333 fmol biotin-labeled 36-bp DNA-fragments derived from the *UPA20* promoter incubated with reduced His_6_::AvrBs3ΔN152(C30S)_Rep_ and His_6_::AvrBs3ΔN152(C30A)_Rep_, respectively (+, incubation with 10 mM DTT overnight). Unlabeled WT and mutant ubm2 DNA fragments (m, [8]) were used as competitor DNA. The experiments were repeated once with similar results.
(EPS)

S5 Fig Expression of AvrBs3, mutant derivatives and Hax2. *Agrobacterium*-mediated expression of 4× c-Myc-tagged AvrBs3 and mutant derivatives, and of Hax2 in leaves of *N*. *benthamiana*. Leaf discs were harvested three dpi from the same areas as investigated in Fig. 4 and analyzed by immunoblot using an α-c-Myc antibody.
(EPS)

S6 Fig Expression of AvrBs3 deletion derivatives in *N*. *benthamiana*. *Agrobacterium*-mediated expression of 4× c-Myc-tagged AvrBs3 WT and deletion constructs in leaves of *N*. *benthamiana*. Samples were harvested three dpi from the same areas as investigated in Fig. 5, and analyzed by immunoblot using an α-c-Myc antibody. The uppermost bands correspond to AvrBs3 and derivatives.
(EPS)

S7 Fig Expression of AvrBs3 and LZm derivatives. **(A)** *Agrobacterium*-mediated expression of 4× c-Myc-tagged AvrBs3 and derivatives in leaves of *N*. *benthamiana*. Leaf discs were harvested three dpi from the same areas as investigated in Fig. 6C and analyzed by immunoblot using an α-c-Myc antibody. **(B)** Expression of FLAG-tagged AvrBs3 and AvrBs3 derivatives in *Xanthomonas*. *Xcv* 85–10 derivatives with the respective plasmids were grown in NYG and analyzed by immunoblot using an α-FLAG antibody. GroEL was used as control for equal loading.
(EPS)

S1 Table Oligonucleotides used in this study. Names, sequences and purpose of the oligonucleotides are given.
(PDF)
