# Supplementary material for: Refined Requirements for Protein Regions Important for Activity of the TALE AvrBs3
Source: PLoS One. 2015 Mar 17;10(3):e0120214. doi: 10.1371/journal.pone.0120214 (PMC4363659; doi:10.1371/journal.pone.0120214)
Supplement: S1 Table — Names, sequences and purpose of the oligonucleotides are given. (PDF) [file pone.0120214.s008.pdf]

Table S1: Oligonucleotides used in this study.

Name	Sequence (5`-3`)	Purpose
Hax-N-F	TTTGGTCTCATATGGATCCCATTCGTTCGCGC	Cloning of *avrBs3* and *hax2* NTR modules
Hax-N-R	TTTGGTCTCAGTTCAGGGGGGCACCCGTCAG	
hax34_C_F	TTTGGTCTCAAGCATTGTTGCCCAGTTATCTC	Cloning of *avrBs3* CTR module (5´ part)
hax2_C_F	TTTGGTCTCAAGCATTTTTGCACAGTTATCTC	Cloning of *hax2* CTR module (5´ part)
hax_C_LZ_rev	TTTGGTCTCGGCGAATGCATGCAAAGAC	Cloning of *avrBs3* and *hax2* CTR modules (5´ part)
hax_C_C_fwd	TTTGGTCTCTTCGCCGATTCGCTGGAGCGTG	Cloning of *avrBs3* and *hax2* CTR modules (3´ part)
hax34_C_R_OS	TTTGGTCTCACACCTGAGGCAATAGCTCCATCA AC	Cloning of *avrBs3* CTR module (3´ part) without stop
hax34_C_R_Stop	TTTGGTCTCAAAGCTTCACTGAGGCAATAGCTC CATC	Cloning of *avrBs3* CTR module (3´ part) with stop
hax2_C_R_Stop	TTTGGTCTCAAAGCTTCAATGAGGCAATAGCT CCATC	Cloning of *hax2* CTR module (3´ part) with stop
Dummy1-6-GW-F	TGGTCTCA*GAAC*AAGTCTTCTGCGGCCGCATTA GGCACCCC	Assembly of repeats 1-6
Dummy1-6-GW-R	AGGTCTCATCTCAAGTCTTCAGGTCGACCTGCA GACTGGCTG	
Dummy7-12-GW-F	TGGTCTCAGAGAAAGTCTTCTGCGGCCGCATT AGGCACCCC	Assembly of repeats 7-12
Dummy7-12-GW-R	AGGTCTCACTGTAAGTCTTCAGGTCGACCTGC AGACTGGCTG	
Dummy13-17,5-GW-F	TGGTCTCAACAGAAGTCTTCTGCGGCCGCATT AGGCACCCC	Assembly of repeats 13-17.5
Dummy13-17,5-GW-R	AGGTCTCATGCTAAGTCTTCAGGTCGACCTGC AGACTGGCTG	
hax_rep_fwd	CACTGACGGGTGGGTCTCTGAACCTGACC	Cloning of *hax2* repeat module
hax_rep_rev	GGGCGAGATAACTGGGGTCTCATGCTCTCCAG	
300ΔAD-CTM-R(WS)	TTTGGTCTCAAAGCTTCACCGCATCACGGTG	Cloning of *avrBs3ΔAD* CTR module
OM_GFP_F	TTTGGTCTCATATGGTGAGCAAGGGCGAGGAG	Cloning of *gfp* with stop
OMS_GFP_R	TTTGGTCTCTAAGCTTCACTTGTACAGCTCGTC CATG	
OM_EV_F	TATGTGATGAATG	Cloning of pGGX1 empty vector control
OM_EV_R	CACCCATTGATCA	
OMS_EV_F	TATGTGATAATAG	Cloning of pGGA2 empty vector control
OMS_EV_R	AAGCCTATTATCA	
Rep-Mut_C30S-F	GTTGCCGGTGCTGTCCCAGGCCC	Mutagenesis of cysteines in single repeats (C30S)
Rep-Mut_C30S-R	GGGCCTGGGACAGCACCGGCAAC	
AR300_C26S_fwd	GCCTTGGCCTCCCTCGGCGGACGTCC	Mutagenesis of cysteines in the CTR
AR300_C26S_rev	GGACGTCCGCCGAGGGAGGCCAAGGC	
Mut_C77S_fwd	GGGTTTTTTCCAGTCCCACTCCCACCC	
Mut_C77S_rev	GGGTGGGAGTGGGACTGGAAAAAACCC	
MutLZ_2/3_fwd	GTTGCCCAGGCCTCTCGCCCTGATCCGGCGGC CGCCGCGTTG	Mutagenesis of imperfect leucine zipper (LZ) motif
MutLZ_2/3_rev	CAACGCGGCGGCCGCCGGATCAGGGCGAGAG GCCTGGGCAAC	
MutLZ_5/6_fwd	CCTTGGCCTGCGCCGGCGGACGTCCTGCGGCG GATGCAGTG	
MutLZ_5/6_rev	CACTGCATCCGCCGCAGGACGTCCGCCGGCGC AGGCCAAGG	
AR_02_KR48/49AA_fwd	TTTGGTCTCTTTGATCGCAGCAACCAATC	Mutagenesis of LZ basic region
delta-N63	TTTGGTCTCATATGGCGTTCTCGGCGGGCAGCT	Cloning of *avrBs3ΔN63*

	TC	NTR module
delta-N92	TTTGGTCTCATATGTTCGGCGCTCACCATACAGAG	Cloning of *avrBs3ΔN92* NTR module
delta-N120	TTTGGTCTCATATGACCATGCGCGTGGCTGTCAC	Cloning of *avrBs3ΔN120* NTR module
300aa153F	TTTGGTCTCATATGGTGGATCTACGCACGCTCG	Cloning of *avrBs3ΔN152* NTR module
BsaI-300aa158F	TTTGGTCTCATATGCTCGGCTACAGCCAGCAGC	Cloning of *avrBs3ΔN158* NTR module
BsaI-300aa163F	TTTGGTCTCATATGCAGCAACAGGAGAAGATCA	Cloning of *avrBs3ΔN163* NTR module
BsaI-300aa168F	TTTGGTCTCATATGATCAAACCGAAGGTTCGTT	Cloning of *avrBs3ΔN168* NTR module
BsaI-300aa173F	TTTGGTCTCATATGCGTTCGACAGTGGCGCAGC	Cloning of *avrBs3ΔN173* NTR module
BsaI-300aa178F	TTTGGTCTCATATGCAGCACCACGAGGCACTGG	Cloning of *avrBs3ΔN178* NTR module
300-CTM-F	TTTGGTCTCATCGCCGATTCGCTGGAGC	Cloning of truncated *avrBs3* CTR modules
300ΔNLSII-AD-CTM-R(WOS)	TTTGGTCTCACACCTGCGGAGGGACCGGTG	Cloning of *avrBs3-C184* CTR module
AR-02-fwd-AvrBs3	TTTGGTCTCTTTGATCAAAAGAACCAATC	Cloning of *avrBs3-C156* CTR module
AR-130aa_R	TTTGGTCTCAGCGACCTGGAGGATACGGTCCCAACGCTGC	Cloning of *avrBs3-C130* CTR module
AR-104aa_R	TTTGGTCTCAGCGACGCGAAAGAGCTGTAACAACCCGTGC	Cloning of *avrBs3-C104* CTR module
AR-91aa_R	TTTGGTCTCAGCGACCTGCGTCATGGCGTCATCAAATG	Cloning of *avrBs3-C91* CTR module
AR-76aa_R	TTTGGTCTCAGCGACCTGGAAAAAACCCAGCACGCGAACC	Cloning of *avrBs3-C76* CTR module
AR-01-rev-AvrBs3	TTTGGTCTCTTCAAGGCCGGCGCGTGCGG	Cloning of *avrBs3-C46* CTR module
SV40-NLS-AR46-F	TTTGGTCTCTTTGATTGCGGAATTAATTCCCGAGC	Cloning of SV40 NLS module
SV40-NLS-AR158-F	TTTGGTCTCTTCGCGGAATTAATTCCCGAGC	
SV40-NLS-R(WOS)	TTTGGTCTCTCACCGGCGGTACCCAATTCGACC	
AvrBs3-AD-Modul_F	TTTGGTCTCAGGTGAACAAGATGAGGACCCCTTCGC	Cloning of AvrBs3 AD module
AvrBs3-AD-Modul_R	AAAGGTCTCTAAGCTTCACTGAGGCAATAGCTC	
